# Assessment of Low-Cost and Higher-End Soil Moisture Sensors across Various Moisture Ranges and Soil Textures

**DOI:** 10.3390/s24185886

**Published:** 2024-09-11

**Authors:** Rajesh Nandi, Dev Shrestha

**Affiliations:** Department of Chemical & Biological Engineering, University of Idaho, Moscow, ID 83844, USA; rajesh@bau.edu.bd

**Keywords:** low-cost sensor, moisture measurement, soil moisture sensor, water management

## Abstract

The accuracy and unit cost of sensors are important factors for a continuous soil moisture monitoring system. This study compares the accuracy of four soil moisture sensors differing in unit costs in coarse-, fine-, and medium-textured soils. The sensor outputs were recorded for the VWC, ranging from 0% to 50%. Low-cost capacitive and resistive sensors were evaluated with and without the external 16-bit analog-to-digital converter ADS1115 to improve their performances without adding much cost. Without ADS1115, using only Arduino’s built-in analog-to-digital converter, the low-cost sensors had a maximum RMSE of 4.79% (*v*/*v*) for resistive sensors and 3.78% for capacitive sensors in medium-textured soil. The addition of ADS1115 showed improved performance of the low-cost sensors, with a maximum RMSE of 2.64% for resistive sensors and 1.87% for capacitive sensors. The higher-end sensors had an RMSE of up to 1.8% for VH400 and up to 0.95% for the 5TM sensor. The RMSE differences between higher-end and low-cost sensors with the use of ADS1115 were not statistically significant.

## 1. Introduction

Maintaining optimal soil moisture is crucial for plant growth and productivity. Soil moisture plays a vital role in various plant processes including nutrient uptake, photosynthesis, and transpiration. Insufficient moisture can hinder plant processes and reduce yields, while excessive moisture may lead to root diseases and increased greenhouse gas emissions, such as nitrous oxide, which contributes to climate change. The soil denitrification rate is positively related to soil water-filled pore space [1]; according to a model, denitrification becomes more important at soil moisture contents greater than 60% water-filled pore space due to a decreased O_2_ supply [2]. N_2_O is a 300 times more potent greenhouse gas than carbon dioxide (CO_2_) over a 100-year time frame [3]. Increased soil N_2_O emissions contribute to climate change. Effective irrigation management avoids under- or overwatering, but it is complex due to varying factors like soil type, climate conditions, soil characteristics, crop species, cultivation practices, and crop needs [4]. According to the 2024 UNESCO report, agriculture accounts for roughly 70% of freshwater withdrawals from freshwater resources [5] and contributes to the freshwater crisis in parts of the world [6]. Irrigation water use is increasing due to the increased demand for agricultural production from population growth [7]. Distributed soil water measurement is required for precision irrigation applications. Understanding the volumetric water content (VWC) of soil is not only important for agriculture production but also for catchment hydrology, flood forecasting, landslide prediction, and other ecosystem services [8]. When it comes to deploying a large number of soil moisture sensors to minimize water waste, the availability of accurate and low-cost soil moisture sensors can be a game changer.

Various methods and sensors are employed to measure the VWC. Traditional VWC measurement using the thermo-gravimetric method is labor-intensive and lacks real-time data. In contrast, modern soil moisture sensors provide real-time measurements and use different techniques, such as neutron thermalization, time domain reflectometry (TDR), time domain transmissometry (TDT), electrical capacitance, and impedance [9]. There is also proximal technology like the cosmic ray neutron sensor (CRNS) which provides estimates of field-averaged soil moisture within a radius of up to 240 m from the sensor [10]. While high-end sensors like TDR offer greater accuracy, they are more expensive, whereas low-cost sensors, resistive and most capacitive types, are more affordable but may be less precise. According to a market research report [11], the global soil moisture sensor market is expected to reach an estimated USD 0.39 billion by 2028 with a compound annual growth rate of 12% from 2023 to 2028. The major drivers for this market are the increasing use of soil moisture sensors in agriculture to enhance productivity and sustain groundwater levels and the growing adoption of smart agricultural equipment globally. Studies indicate that sensor performance can vary with soil type and calibration, making it essential to select the appropriate sensor and calibrate it properly for accurate irrigation management.

Current research in in situ soil moisture measurement focuses on evaluating and improving the accuracy of various sensors used to monitor the VWC. Traditional methods like thermo-gravimetric analysis are time-consuming and laborious. Modern sensors such as neutron thermalization, TDR, and TDT are quick and accurate but costly. Research indicates that low-cost sensors, such as resistive and most capacitive types, may be less precise. Low-cost sensors’ accuracy may vary depending on soil textures [12] and inherent variation in sensor impedances. Selecting a sensor for a specific soil type is important for proper irrigation scheduling. Singh et al. [13] evaluated the performance of six commercial sensors, and Kargas and Soulis [14] investigated the performance of the CS655 soil moisture sensor under ten different soil conditions and showed that sensor performance varied in different soil conditions. Dong et al. [15] used EC5 and CS616 sensors under three different soil conditions and found that the sensors did not work satisfactorily in all soil types. Ferrarezi et al. [16] evaluated the performance of various types of soil moisture sensors in sandy soil in Florida and found that some sensors performed well with factory-based calibration and some sensors required soil-specific calibration. They concluded that soil-specific calibration improves the measuring accuracy significantly.

We hypothesize that the precision and accuracy of low-cost sensors can be enhanced by utilizing a higher-bit-depth analog-to-digital converter in conjunction with a low-pass analog filter. The purpose of this study is to test the hypothesis by comparing the accuracy and precision of low-cost (lower-end) and high-cost (higher-end) soil moisture sensors in measuring the VWC with and without the external chip. Four different sensors were compared. A low-cost resistive soil moisture sensor (FC-28), a generic capacitive soil moisture sensor (v.1.2), an intermediate-cost capacitance-based soil moisture sensor (VH400), and a higher-end soil moisture sensor (5TM) (www.metergroup.com) also based on capacitive sensing were used.

## 2. Materials and Methods

### 2.1. Soil Moisture Sensors

Four sensors were selected for this research based on their cost: a low-cost resistive sensor, a low-cost capacitive sensor, an intermediate-cost VH400 (Vegetronix, Sandy, UT, USA) sensor, and a high-cost 5TM sensor (Decagon, Pullman, WA, USA). A brief description of each sensor is provided in the following subsections.

#### 2.1.1. Low-Cost Resistive Sensor

**Figure 1:**
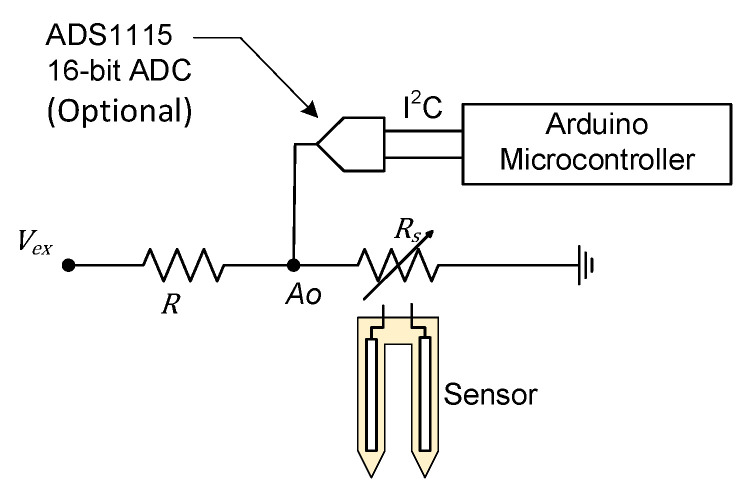
FC-28 resistive sensor circuit diagram ($V_{ex}$ = 5 V and R = 10 kΩ. *R_s_* represents the actual sensor. Analog output *Ao* can be either directly read from a microcontroller’s analog-to-digital converter (ADC) or read digitally if an external ADC is used).

Resistive sensors measure the soil moisture by correlating electrical resistance to water content. They have two exposed probes which are placed directly into the soil (Figure 1). For this study, an FC-28 resistive sensor (Shenzhen Jiexing Weiye Electronic Co., Ltd., Shenzhen, China) was used to measure the VWC. The product description claims the sensor is made of corrosion-resistant materials for a long service life, but the exact material composition is not specified.

The sensor measures the resistance between the probes by sending an electrical current. The higher the VWC of the soil, the lower the resistance. The resistance change translates to the voltage drop across the probes, which is determined by measuring voltage $A_{o}$ in Figure 1, and is given by the following:

(1) $$A_{o}=V_{ex}\frac{R_{s}}{R_{s}+R}$$

where $V_{ex}$ is the excitation voltage, and $R_{s}$ and R are the resistances, as shown in Figure 1. The sensor’s output is an analog voltage that varies nonlinearly to the soil’s VWC. A microcontroller can be used to read the voltage. This type of sensor costs around USD 1.50 per unit.

#### 2.1.2. Low-Cost Capacitive Sensor

Capacitive soil moisture sensors measure a change in the capacitance to determine the VWC. These sensors are commonly built with two parallel plates separated by a dielectric medium. The dielectric constant varies with the VWC. A low VWC in the soil corresponds to a low dielectric constant and so is the capacitance between the plates. If the VWC in the soil increases, the dielectric constant also increases, and consequently, the capacitance between the two plates also increases. A capacitive soil moisture sensor (version 1.2) (www.dfrobot.com) was used in this study. 

**Figure 2:**
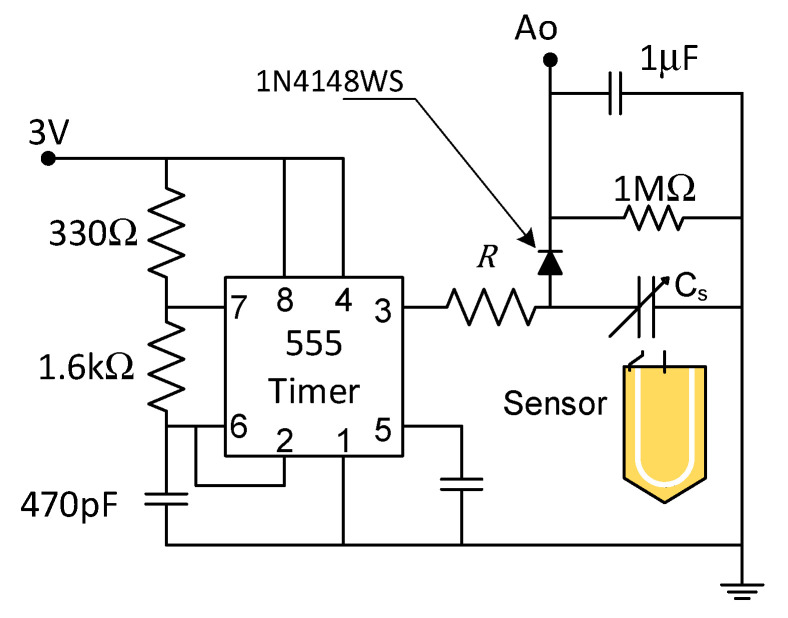
The capacitive soil probe circuit diagram. R = 10 kΩ. Adapted from [17]. Physical pin numbers are indicated on 555 Timer.

The capacitor plate was excited with an astable 555 timer with 3 V square wave at 870 kHz. The generic sensor had a built-in voltage regulator that regulated the supply voltage between 3.3 and 5.5 V down to 3 V and a 555 timer in a stable configuration (Figure 2).

By coupling the sensor with a timer circuit that produces digital pulses with frequency f, we obtain an analog voltage ($A_{o}$) that an Arduino board can read, which is given by the following:

(2) $$A_{o}\approx \left(\frac{3}{\sqrt{1+{\left(2\pi fRC_{s}\right)}^{2}}}-0.07\right)\;V$$

R is 10 kΩ and $C_{s}$ is the sensor capacitance (Figure 2). This voltage can then be converted to the VWC. This type of sensor costs about USD 2.00 per unit or less in bulk. 

#### 2.1.3. VH400 Moisture Sensor

The VH400 series (Vegetronix, Sandy, UT, USA) soil moisture sensor probes are based on capacitance, with two electrodes creating a capacitor that allows the electric field to penetrate the surrounding soil. The sensor uses an unbalanced design with one electrode generating the electric field and the second electrode internally fixed to ground potential. Stray capacitances can affect the measurement result, which can be reduced by shielding the cable. The circuit diagram of this sensor is proprietary and not published. Standard cable lengths for the probes are 2 m, 5 m, and 10 m long. The price of a VH400 with a 2 m cable is around USD 42.00.

#### 2.1.4. 5TM Moisture Sensor

The Decagon 5TM sensor (Decagon, Pullman, WA, USA) has an accuracy of ±0.03 m^3^/m^3^ (±3%) with a measurement range of 0–1 m^3^/m^3^. The electrical conductivity and temperature in soils can also be monitored using this sensor. Each of the three measurements is performed separately. Using capacitance/frequency domain technology, this sensor measures the media’s dielectric constant to determine the VWC. The 5TM sensor measures the dielectric permittivity of the surrounding medium using an electromagnetic field. The sensor uses 70 MHz frequency, with a claim that this range reduces the impacts of salt and soil texture, enhancing the sensor’s accuracy in the majority of soils. A thermistor is integrated into the probe for temperature measurement, and the use of three prongs allows for the measurement of resistivity and capacitance simultaneously. The cost of a 5TM sensor is around USD 350.00. The VWC is derived from the Topp equation [18].

(3) $$\theta =4.3\times 10^{-6}\times \varepsilon^{3}-5.5\times 10^{-4}\times \varepsilon^{2}+{2.92\times 10}^{-2}\times \varepsilon -5.3\times 10^{-2}$$

where θ is the VWC and ε is the relative dielectric permittivity to air. The Topp equation results in measurements within 3% VWC of the actual soil VWC for mineral soil.

### 2.2. Signal Stability Improvement Using an ADC

**Figure 3:**
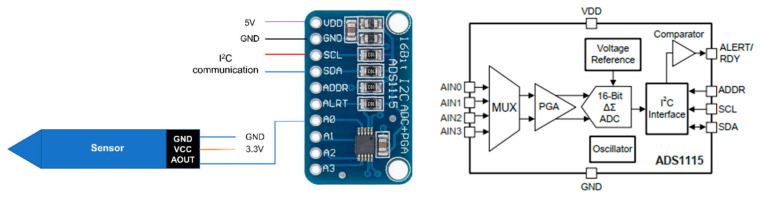
Sensor connected to ADS1115 breakout board (**left**) and ADS1115 block diagram (**right**), adapted from the user manual of ADS1115 [19]. Only one channel was used with unity gain. Reprinted with permission courtesy of Texas Instruments.

To increase the analog-to-digital conversion resolution and to filter the high-frequency noise, we used an external 16-bit ADC (Texas Instruments’ ADS1115). ADS1115 is a 16-bit, low-power, I2C-compatible analog-to-digital converter that includes an oscillator and a low-drift voltage reference. The ADS1115 breakout board costs about USD 5.00 apiece. In addition to ADC functionality, the chip consists of a low-pass filter, a programmable gain amplifier (PGA), and a programmable data rate. Furthermore, the ADS1115 model has a multiplexer that can measure either two differentials or four single-ended inputs. A block diagram of ADS1115 is shown in Figure 3 with the circuit diagram for the connection.

Arduino is a trademarked yet open architecture family of microcontrollers. Arduino Nano, one of its models, features an ATmega328P microprocessor (Microchip, Chandler, AZ, USA), equipped with eight multiplexed 10-bit analog-to-digital converters, labeled A0 through A7. The microcontroller has an ADC noise reduction mode that stops the CPU and all I/O modules except the asynchronous timer and ADC to minimize switching noise during ADC conversions. In contrast, ADS1115 utilizes a delta–sigma analog-to-digital converter. Delta–sigma ADCs offer low noise, are tolerant to a wide range of frequencies, and provide a more stable high-resolution output compared to the successive approximation ADC used in the Nano. 

### 2.3. Sample Collection and Preparation

An aggregate soil sample was collected from the University of Idaho’s Parker Farm (latitude 46.723099, longitude −116.962002). The soil is dark brown silty loam. This is a typical soil found in the Inland Northwest region of the United States, commonly referred to as the Palouse Series Soil. The soil taxonomic class is “Fine-silty, mixed, superactive, mesic Pachic Ultic Haploxerolls” [20]. The soil textural analysis showed 26% sand, 58% silt, and 16% clay. 

**Figure 4:**
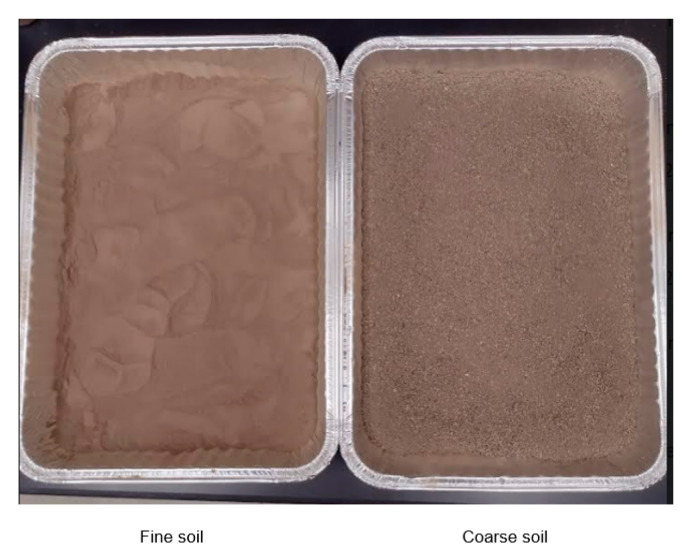
The fine (**left**) and coarse (**right**) soil used in this study.

The collected soil sample was ground and oven dried at 105 °C following the ANSI/ASABE S633 standard [21] until a constant weight was observed which took about 24 h. The dried soil was then separated into coarse and fine fractions using 1.981 mm and 0.147 mm sieves. The coarse soil represents soil particles of sizes between 1.981 mm and 0.147 mm and fine soil represents a soil size of less than 0.147 mm. The demarcation was selected around particle size for sand which is from 2 mm to 0.2 mm. Fine and coarse soils were mixed at a 1:1 ratio (by weight) and thoroughly mixed to prepare a medium-textured soil. Figure 4 shows the visual difference between the fine- and coarse-textured soils used in this study.

### 2.4. Measurement of Soil Bulk Density

Soil bulk density was measured in the laboratory from a disturbed sample. The soil was oven-dried at 105 °C for 24 h and placed into a container of known volume, and the mass of the soil was recorded using a weighing scale. The container was filled while gently shaking, and physical compaction was not used. Variability in bulk density measurement is unavoidable; however, we expect that the variability in bulk density measurement is random error and reduced when averaged from replicates. The bulk density was determined by dividing the mass by the volume of the soil. The bulk density and standard deviation (SD) of course-, fine-, and medium-textured soil was 1.11 (SD = 0.02), 1.07 (SD = 0.02), and 1.26 (SD = 0.04) g/cm^3^, respectively. 

### 2.5. Experimental Setup 

**Figure 5:**
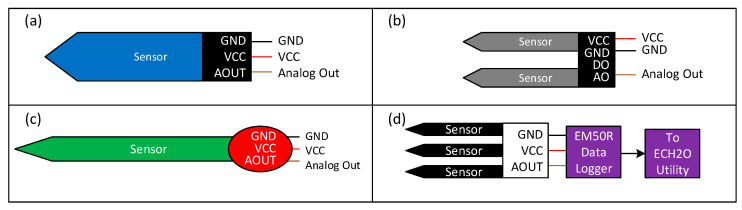
Circuit connection diagram of (**a**) low-cost capacitive sensor (v.1.2), (**b**) FC-28 low-cost resistive sensor, (**c**) VH400 sensor, and (**d**) 5TM sensor.

The circuit connection diagram using different sensors is shown in Figure 5. One of the capacitive, resistive, or VH400 sensors was connected to the analog input A0 pin of Arduino Nano. The data were transmitted to the host computer connected to the Arduino Nano via RS232 serial communication. All experiments were conducted at a room temperature of 20 °C.

**Figure 6:**
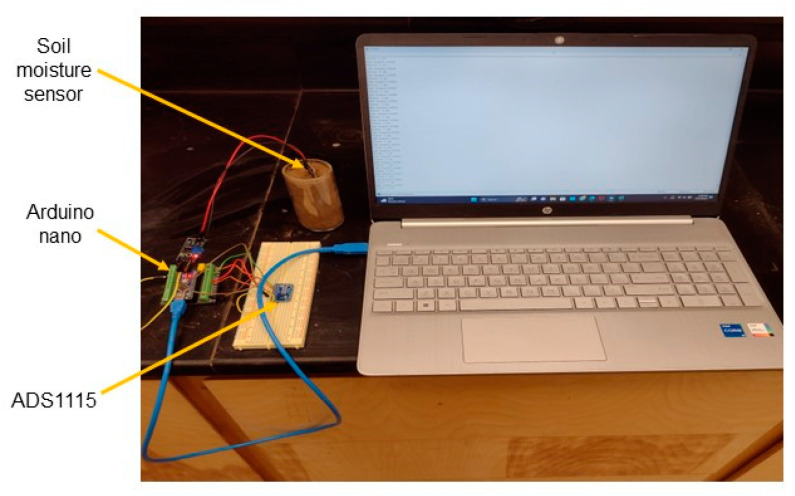
Experimental setup for this study with ADS1115.

The 5TM sensor was directly read from an Em50R data logger [22]. The processed data from the signal processing unit were received in a computer using RS232 serial communication or the ECH20 utility (Decagon, Pullman, WA, USA) for the Em50R data logger. Figure 6 shows the experimental setup used in this study.

### 2.6. Experimental Procedure

Three different soil textures were used to assess the accuracy of four soil moisture sensors. A calculated amount of water by volume was added to dry soil to prepare 0%, 10%, 20%, 30%, 40%, and 50% of soil VWC. The water volume was measured using a 50 mL burette (±0.1 mL), and the soil mass was measured using a digital balance with an accuracy of 0.01 g (Sartorius laboratory balance, Model L2200S, BioArrow Technology Ltd., Göttingen, Germany). To maintain homogeneity, the calculated amount of water was added incrementally to the dry soil, and the soil was thoroughly mixed to achieve a consistent distribution of moisture throughout the soil sample. The soil was manually compacted as tightly and uniformly as possible to ensure optimal sensor contact and to prevent the formation of large voids. This process was repeated for each targeted VWC level. The sensors were then inserted into the soil to measure the VWC. The analog sensor output value was recorded against the VWC. For measurement using the 5TM sensor, an EM50R data logger was used with the ECH2O utility, where the output was the percentage VWC. Each experiment with a combination of soil and moisture was replicated three times for the same input to evaluate the sensor’s precision. More than 100 repeated measurements were taken within each replication to calculate the statistics.

### 2.7. Data Analysis

The sensor performances were evaluated for measurement accuracy and precision. The closer the measured values are, the higher the sensor precision. The difference between the actual and the measured values represents accuracy. A third-order polynomial was fitted to obtain calibration curves for the selected sensors in fine and coarse soils using Microsoft Excel’s Data analysis tool pack (Office 360-2024 version, Microsoft, Redmond, WA, USA). A third-order polynomial was chosen to be consistent with the 5TM sensor which uses Equation (3). A good sensor should have a high accuracy and a high precision. An ANOVA on the Root Mean Squared Error (RMSE) was performed to find the statistical significance of differences in sensor performance among soil groups.

## 3. Results

### 3.1. Performance Comparison without ADS1115

**Figure 7:**
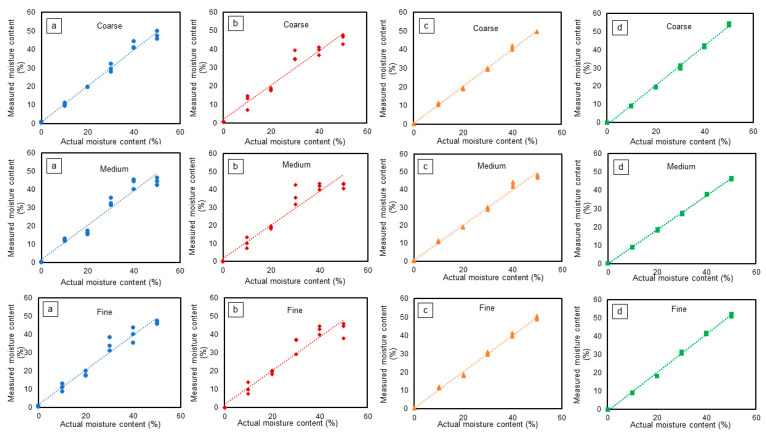
Calibration curve for sensors in coarse, medium, and fine soil: (**a**) capacitive, (**b**) resistive, (**c**) VH400, and (**d**) 5TM, each color representing a specific sensor. Each experiment was conducted with three replications.

Figure 7 shows the actual versus predicted moisture content for various soil textures. From the figure, it is evident that without additional signal processing, the sensor output exhibited greater deviations for the low-cost capacitive and resistive sensors, whereas the VH400 and 5TM sensors consistently demonstrated a more precise output. This observation underscores the notable differences in precision among the sensors which may be stemming from the digital noise of the internal ADC of the Arduino board. With no modifications, the VH400 and 5TM sensors exhibited more consistent readings compared to the low-cost capacitive and resistive sensors. Table 1 shows the results of regression analysis including different statistical parameters for sensor performance in different types of soil texture. For coarse soil, the resistive sensor had the lowest R^2^ values for the linear regression line between the actual and predicted VWC (Table 1). The VH400 and 5TM sensors showed a higher R^2^. The higher the value of R^2^, the greater the degree of fit of the model to the data; however, it does not indicate whether the estimates and predictions are biased or not [23].

The result of this study agrees with Okasha et al. [24] who obtained R^2^ values for low-cost capacitive sensors from 0.96 to 0.99. Adla et al. [9] reported an R^2^ value of 0.89 with a first-order linear line in sandy soil for low-cost resistive sensors similar to the one used in this research. Praštalo et al. [25] evaluated low-cost capacitive and resistive sensors in gravel and sandy soil with linear fit regression R^2^ values of 0.9036 and 0.9023, respectively. The difference between the current study and the previous studies may be due to the difference in sensor models, the order of polynomials (first- vs. third-order polynomial) used to fit the data, and experimental conditions. 

The RMSE values for the capacitive, resistive, VH400, and 5TM sensors were 1.88%, 3.83%, 0.85%, and 0.82% VWC, respectively (Table 1). The RMSE is a measure of accuracy; therefore, the VH400 and 5TM sensors showed less error in estimating the VWC than the capacitive and resistive sensors. The SD for the capacitive, resistive, VH400, and 5TM sensors was found to be 1.43%, 2.06%, 0.51%, and 0.34% VWC, respectively. The SD is a measure of precision. The lower the value of the SD, the higher the precision. The VH400 and 5TM sensors showed higher precision than the capacitive and resistive sensors. ANOVA showed that the capacitive and resistive sensors’ error was significantly higher than that of the VH400 and 5TM sensors (*p* = 0.007). 

The resistive sensor had the lowest R^2^ of 0.93 compared to the VH400 and 5TM sensors at 0.995 and 0.997. The generic capacitive sensor had an intermediate R^2^ value of 0.96. The RMSE and SD values for the capacitive and resistive sensors were significantly higher compared to the VH400 and 5TM sensors (*p* = 0.007). Therefore, the precision of the VH400 and 5TM sensors was also higher in fine soil. 

In medium-textured soil, a similar trend was observed as in coarse and fine soils. The 5TM sensor showed a better fit with an R^2^ value of 1.0 followed by the VH400 (R^2^ = 0.99), capacitive (R^2^ = 0.95), and resistive (R^2^ = 0.92) sensors. The results of the current study are in agreement with Praštalo et al. [25], who showed an R^2^ value of 0.9856 using the VH400 sensor. The R^2^ value for the 5TM sensor was lower (0.95) in a previous study by Li et al. [26] and improved with a linear calibration model (0.99). 

Comparing the actual VWC and predicted value (Figure 7), a simple capacitive and resistive soil moisture sensor with no intermediate A/D converter frequently overestimated or underestimated the VWC. The RMSE for the resistive sensor was highest at 4.8%. For instance, at 50% moisture content, the predicted value of the capacitive moisture sensor was between 42.52% and 46.43% in medium-textured soil. The resistive sensor, on the other hand, predicted between 40.5% and 43.40%. Overall, the capacitive soil moisture sensor had ±0.09% to 4.68%, 0.08% to 7.48%, and 0.08% to 8.54% error in coarse-, medium-, and fine-textured soils, respectively. Conversely, the resistive soil moisture sensor overestimated or underestimated the VWC in coarse-, medium-, and fine-textured soils by up to 9.35%, 12.47%, and 11.97%, respectively. However, the VH400 and 5TM sensors showed close values to the actual ones. The maximum difference between the actual VWC and the VH400 predicted value was 4.31%. The 5TM sensor overestimated or underestimated the VWC by up to 4.23%. The specification for the 5TM sensor’s accuracy was ±3% VWC in mineral soils with an electrical conductivity of less than 10 dS/m. However, the specifications are usually provided for a 95% confidence interval or RMSE, and some data points are expected to be outside of the 95% confidence interval. It should be noted that the error of 4.23% was the extreme we observed.

The discrepancy in the performance of the simple resistive and capacitive sensors in different soil textures could be attributed to insufficient soil contact. The accuracy of the VWC measurement depends on the sensor installation technique. The installation should be conducted carefully because the soil moisture sensor only detects a tiny amount of the soil surrounding the sensor. Many previous studies [27,28,29,30] highlighted that the sensor and soil must make good contact in order to prevent an air gap. However, the error of estimation in fine, coarse, and medium soil textures was not significantly different (*p* = 0.21). The results of this study suggest that the VH400 and 5TM sensors performed better than the simple resistive and capacitive sensors.

### 3.2. Performance Comparison with ADS1115

The use of ADS1115 provided more stable readings and improved the accuracy for low-cost sensors. The R^2^ value using ADS1115 with the capacitive sensor for all soil types was around 0.99 (Table 1). The R^2^ value using ADS1115 with the resistive sensor for coarse-, medium-, and fine-textured soils was 0.99, 0.98, and 1.0, respectively. The capacitive sensor showed an R^2^ value of 0.99 for all types of soil textures. It is evident from Table 1 that the addition of ADS1115 improved the R^2^ values of both the low-cost sensors. The RMSE values from the capacitive and resistive sensors also reduced with ADS1115. The RMSE values with ADS1115 were between 1.27% and 1.87% moisture content for the capacitive sensor and between 0.64% and 2.64% VWC for the resistive sensor.

It is important to note that the results presented in Table 1 pertain to soil at 20 °C. Since both the resistivity and dielectric constant of water vary with temperature, soil moisture measurements taken across different temperatures require corrections to account for these changes, especially if such corrections have not already been applied, as is often the case with low-cost sensors.

The SD for the capacitive sensor was 0.10%, 0.06%, and 0.03% of the measured VWC for coarse-, medium-, and fine-textured soils. On the other hand, the resistive sensor showed an SD of 0.09%, 0.14%, and 0.04% for coarse-, medium-, and fine-textured soils, respectively. The addition of ADS1115 lowered the SD and improved the R^2^. ANOVA showed that the predicted SD using ADS1115 was significantly lower for both the resistive and capacitive sensors (*p* = 0.00) compared to using the built-in Arduino ADC.

**Figure 8:**
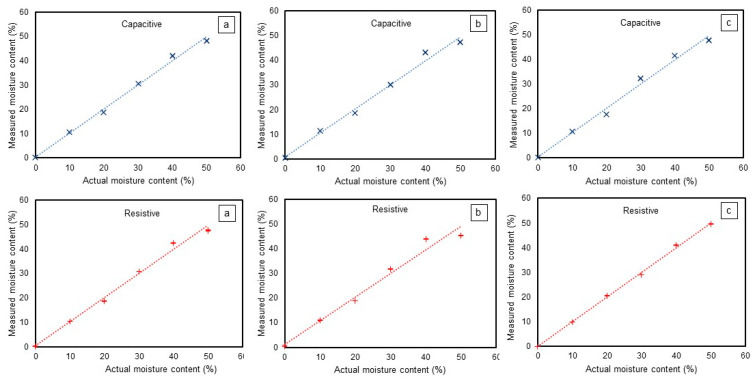
Calibration curve using ADS1115 with capacitive and resistive sensors, also differentiated by color in (**a**) coarse-, (**b**) fine-, and (**c**) medium-textured soil. Each experiment was conducted with three replications.

The measured vs. predicted VWC with the capacitive and resistive sensors with the addition of ADS1115 is shown in Figure 8. With ADS1115, both the capacitive and resistive sensors showed a consistent output. The capacitive sensor showed a deviation of up to 2.16%, 3.2%, and 2.47% of VWC in coarse-, medium-, and fine-textured soils, respectively. The deviation in the resistive sensor’s predicted VWC value in coarse-, medium-, and fine-textured soils was up to 2.6%, 4.73%, and 1.06%, respectively, with ADS1115. Therefore, a significant precision improvement in the low-cost capacitive and resistive sensors was observed with ADS1115.

The average voltage fluctuation using the low-cost capacitive sensor in fine-textured soil with a constant VWC was around 27 mV using the Arduino A/D converter. The addition of ADS1115 reduced the signal fluctuation to only 0.95 mV. Similarly, the generic resistive sensor showed an average fluctuation of 59 mV, and the addition of ADS1115 reduced the fluctuations to 0.77 mV. The significant reduction in the voltage fluctuation observed after adding ADS1115 indicates that ADS1115 effectively improves the stability and accuracy of the analog measurements. The high fluctuations in voltage observed with the low-cost capacitive and resistive soil moisture sensors likely include noise from various sources such as electrical interference, sensor imperfections, and environmental factors. ADS1115′s higher resolution and potential filtering capabilities help reduce the impact of noise on the sensor readings, resulting in much lower voltage fluctuations.

**Figure 9:**
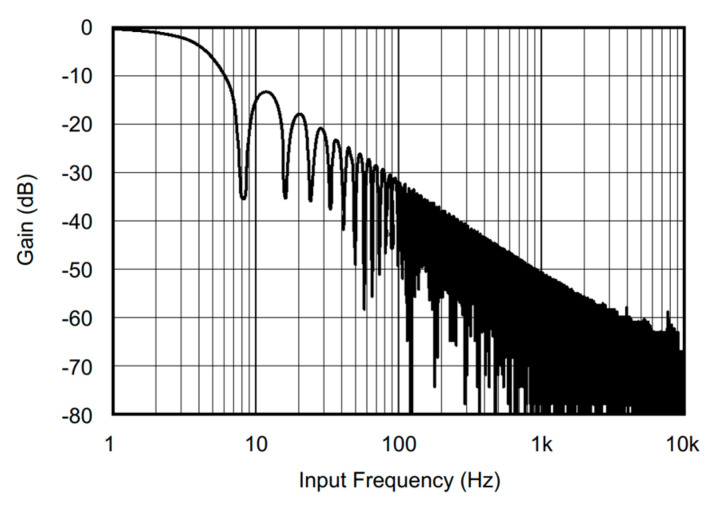
ADS1115 frequency response at 8 samples per second data rate [19]. Reprinted with permission courtesy of Texas Instruments.

One possible reason for why ADS1115 improved the predictive performance could be its built-in low-pass filter, which acts as an anti-alias filter to reduce high-frequency noise and interference in the analog signal. The filter limits the impact of electrical noise on measurements prior to converting the signal to a digital value. The filter had a cutoff frequency of about 5 Hz (read from Figure 9 at −3.01 dB gain). Thus, the analog input filter contributes to a more stable analog signal by filtering away high-frequency noise. Conversely, without ADS1115, the inconsistency in sensor readings may have originated from digital noise in the internal ADC of the Arduino board. Another factor that could have contributed to improved performance is the bit resolution. The Arduino Nano has a 10-bit ADC with an absolute accuracy of 2 Least Significant Bit (LSB). For 0–5 V conversion at 10 bit, a 2-bit error corresponds to an error of 9.8 mV. ADS1115 has a 16-bit ADC resolution, which provides 64 times higher resolution at 0.16 mV than a 10-bit ADC.

One of the important considerations that determine the choice of sensors by commercial growers is the cost, particularly if the field with significant spatial variability [31]. The cost of the resistive and capacitive sensors is relatively lower than that of the VH 400 and 5TM sensors. Therefore, comparing the costs, the resistive and capacitive sensors with ADS1115 could be a good choice for a soil moisture monitoring system. Further study needs to be conducted to evaluate the long-term durability and reliability of these low-cost sensors in real-world field conditions.

## 4. Conclusions

The VH400 and 5TM sensors showed better precision and accuracy than the low-cost capacitive and resistive sensors with direct analog output measurement using Arduino’s analog-to-digital converter. The low-cost sensors exhibited a higher signal fluctuation and offset when connected directly to the Arduino microcontroller. The addition of an external high-bit-resolution analog-to-digital converter (ADS1115) with a built-in low-pass filter significantly enhanced the prediction performance of these low-cost sensors. Specifically, the RMSE for the resistive sensor reduced from 4.79% to 2.64%, and the standard deviation of measurements reduced from 2.58% to 0.14%. Similarly, for the capacitive sensor, the RMSE was reduced from 3.87% to 1.87%, with the standard deviation decreasing from 2.24% to 0.10%. Notably, the difference in the RMSE for the low-cost and high-end sensors with ADS1115 was statistically insignificant. 

Therefore, low-cost sensors, when paired with affordable analog-to-digital converters like ADS1115, offer a viable and cost-effective alternative for soil moisture measurement in the field, providing performance comparable to that of more expensive options. Furthermore, the sensors demonstrated consistent performance across different soil textures with no significant variation. However, it is important to note that the testing of low-cost sensors in this research was limited to a short-term evaluation. Long-term sensor performance remains an area for future investigation.

## Figures and Tables

**Table 1:** Results of regression analysis (RMSE and SD are % VWC).

Soil Texture	Sensor Type	R^2^	RMSE	SD
Coarse	Capacitive	0.99	1.88	1.43
	Capacitive with ADS1115	0.99	1.27	0.10
	Resistive	0.95	3.83	2.06
	Resistive with ADS1115	0.99	1.55	0.09
	VH400	1.00	0.85	0.51
	5TM	1.00	0.82	0.34
Fine	Capacitive	0.96	3.22	2.24
	Capacitive with ADS1115	0.99	1.81	0.08
	Resistive	0.93	4.39	2.59
	Resistive with ADS1115	1.00	0.64	0.04
	VH400	1.00	1.17	0.71
	5TM	1.00	0.95	0.29
Medium	Capacitive	0.95	3.78	1.48
	Capacitive with ADS1115	0.99	1.87	0.06
	Resistive	0.92	4.79	2.07
	Resistive with ADS1115	0.98	2.64	0.14
	VH400	0.99	1.80	0.69
	5TM	1.00	0.47	0.28

## Data Availability

Data can be made available upon request.
